# Immune cell profiles of patients with interstitial cystitis/bladder pain syndrome

**DOI:** 10.1186/s12967-022-03236-7

**Published:** 2022-02-21

**Authors:** Robert M. Moldwin, Vishaan Nursey, Oksana Yaskiv, Siddhartha Dalvi, Eric J. Macdonald, Michael Funaro, Chengliang Zhang, William DeGouveia, Marina Ruzimovsky, Horacio R. Rilo, Edmund J. Miller, Souhel Najjar, Inna Tabansky, Joel N. H. Stern

**Affiliations:** 1 The Smith Institute for Urology, Northwell Health, 450 Lakeville Road New Hyde Park, Lake Success, NY USA; 2 Department of Urology, Donald and Barbara Zucker School of Medicine at Hofstra/Northwell, 500 Hofstra University Blvd, Hempstead, NY USA; 3 Department of Neurology, Donald and Barbara Zucker School of Medicine at Hofstra/Northwell, Hempstead, NY USA; 4 Institute of Molecular Medicine, The Feinstein Institutes for Medical Research, Manhasset, NY USA; 5 Department of Pathology, Donald and Barbara Zucker School of Medicine at Hofstra/Northwell, Hempstead, NY USA; 6 Department of Surgery, Donald and Barbara Zucker School of Medicine at Hofstra/Northwell, Hempstead, NY USA; 7 RDS2 Solutions, Stony Brook, NY USA; 8 Department of Neurobiology and Behavior, The Rockefeller University, New York, NY USA; 9 Department of Neurology, Lenox Hill Hospital, New York, NY USA; 10 Renaissance School of Medicine, Stony Brook University, Stony Brook, NY USA

## Abstract

Interstitial cystitis/bladder pain syndrome (IC/BPS) is a disorder characterized by bladder pain upon filling which severely affects quality of life. Clinical presentation can vary. Local inflammatory events typify the clinical presentation of IC/BPS patients with Hunner lesions (IC/BPS-HL). It has previously been proposed that B cells are more prevalent in HL, but understanding their exact role in this environment requires a more complete immunological profile of HL. We characterized immunological dysfunction specifically in HL using immunohistochemistry. We detected significantly more plasma cells (50× increase, p < 0.0001), B cells (28× increase, p < 0.0001), T cells (3× increase, p < 0.0001), monocytes/macrophages (6× increase, p < 0.0001), granulocytes (4× increase, p < 0.0001), and natural killer cells (2× increase, p = 0.0249) in IC/BPS patients with HL than in unaffected controls (UC). Patients with IC/BPS-HL also had significantly elevated urinary levels of IL-6 (p = 0.0054), TNF-α (p = 0.0064) and IL-13 (p = 0.0304) compared to patients with IC/BPS without HL (IC/BPS-NHL). In contrast, IL-12p70 levels were significantly lower in the patients with HL than in those without these lesions (p = 0.0422). Different cytokines were elevated in the urine of IC/BPS patients with and without HL, indicating that different disease processes are active in IC/BPS patients with and without HL. Elevated levels of CD138+, CD20+, and CD3+ cells in HL are consistent B and T-cell involvement in disease processes within HL.

**Supplementary Information:**

The online version contains supplementary material available at 10.1186/s12967-022-03236-7.

## Introduction

Interstitial Cystitis/Bladder Pain Syndrome (IC/BPS) affects millions of individuals within the US [1]. It is defined by bladder pain/discomfort accompanied by irritative voiding symptoms in the absence of other identifiable etiologies. IC/BPS is often difficult to diagnose because symptoms are similar to other disorders, such as overactive bladder, vulvodynia, endometriosis, and prostatitis [2]. Identification of targets for diagnostic tests and treatments that consistently and accurately predict disease status and/or provide insights into disease mechanisms is an ongoing challenge [3].

**Fig. 1:**
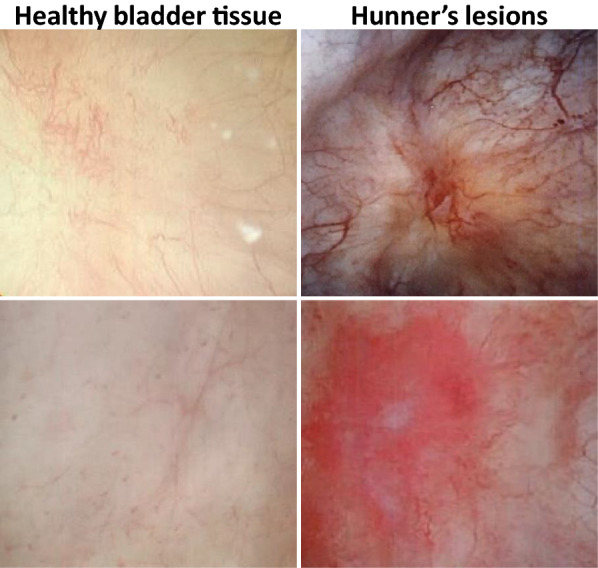
Cystoscopic images of Hunner Lesions (HL) from 2 different patients with Interstitial Cystitis/Bladder Pain Syndrome with Hunner Lesions (IC/BPS-HL) compared to healthy bladder tissue. HLs are typically circumscribed, reddened mucosal areas with small vessels radiating towards a central scar and are seen in about 5 to 10 percent of patients with IC/BPS [4]. Diagnosis is made via cystoscopy and confirmed by biopsy

While most patients with IC/BPS show no cystoscopic abnormalities of the bladder wall (IC/BPS-NHL), a small percentage of patients with IC/BPS present with focal regions of gross bladder inflammation termed Hunner lesions (HL). These lesions are typically associated with small vessels radiating toward a central scar and a fibrinous exudate (Fig. 1) [4]. Although all patients afflicted with IC/BPS present with a similar clinical presentation, those with IC/BPS-HL are believed to be a more homogeneous group due to the clear demonstration of inflammatory disease. A question that has significant clinical ramifications is whether IC/BPS with Hunner lesions (IC/BPS-HL) represents a point on the spectrum of IC/BPS, or a separate disease process altogether. Determining which cytokines and immunological pathways are present in HL could help identify whether it is part of the spectrum of IC/BPS-NHL or a unique disease.

Evidence for immunologic dysfunction in IC/BPS includes the observation that several autoimmune disorders are significantly more prevalent in patients with IC/BPS than in the general population. These disorders include: Sjögren’s syndrome, which has been found in 7.2% of patients with IC/BPS as opposed 0.5% in the overall population [5] and rheumatoid arthritis, which has been observed in 4–13% of patients with IC/BPS compared to 1.0% of the general population [6].

Immune system involvement in IC/BPS is also supported by the elevated counts of immune cells found in the bladder of patients with IC/BPS. Elevated mast cell counts have been reported in the submucosal and detrusor layers of the bladder wall of patients with IC/BPS-HL and IC/BPS-NHL [7]. Mast cells can produce and release numerous pro inflammatory mediators including Interleukin-6 (IL-6), Interleukin-8 (IL-8), prostaglandins, histamine, and Vascular Endothelial Growth Factor (VEG-F) [8]. Previous studies have shown elevated levels of IL-6 and IL-8 in the urine of patients with IC/BPS, and have also linked urinary VEG-F levels with higher rates of IC/BPS bladder glomerulations [9, 10].

The majority of studies in the current literature have focused either on soft tissue or urine analysis from generalized IC/BPS patient cohorts. While investigators have identified infiltrating immune cells in the bladder and elevated concentrations of multiple cytokines and antibodies in the urine [1, 11, 12], results have been disparate [9, 13], possibly owing to inconsistent disease classifications between treatment centers.

Evaluation of bladder biopsies of the more homogeneous population of IC/BPS-HL patients, have shown expression of several B and T cell markers including Cluster of Differentiation 20 (CD20), and CD79A [14]. Other studies also confirmed significant elevation in CD138 positive (CD138+) plasma cell counts within the bladder of patients with IC/BPS-HL. Substantial plasma cell inflammation (200+ cells/mm^2^) was observed in 93% of IC/BPS-HL samples and 8% of IC/BPS-NHL samples [15].

While immune cell infiltrates and inflammatory mediators appear to play an important role in the pathogenesis of IC/BPS-HL, further elucidation of differences between IC/BPS subgroups and control populations is needed. Our study examined immune cells and associated molecules in subpopulations of IC/BPS patients as assessed through urine and bladder biopsies. We used immunohistochemistry to create bladder immune cell profiles of patients with IC/BPS-HL and mesoscale discovery cytokine assays detect differences in urinary cytokine concentrations between IC/BPS-HL, IC/BPS-NHL, and unaffected control (UC) sub-populations.

## Materials and methods

### Tissue acquisition

All aspects of the study protocol received full board review and approval by the Institutional Review Board at Northwell Health. Informed consent was obtained from all research participants prior to sample acquisition and health information retrieval. The study approval number is 17-0254-NSUH.

Cold cup biopsy samples were obtained from 48 patients with IC/BPS-HL, fixed in 10% formalin, and sent for immunohistochemistry (described below). Hunner lesions were defined as regions of focal inflammation that were present upon cystoscopy without the need for hydrodistention. Biopsy samples from patients with IC/BPS-NHL were not obtained because patients without Hunner lesions rarely undergo biopsy at our Center. IRB stipulations did not allow for biopsy collection if a biopsy was not already scheduled as standard of care. Biopsy samples from 2 patients who presented with benign bladder tumors and no morphologic or clinical features of IC/BPS or any other urological malignancy were also retrieved to serve as unaffected controls (UC).

Urine samples were obtained from 18 patients with IC/BPS-HL, 18 with IC/BPS-NHL and 4 UC. These samples were obtained from a separate patient cohort than the one whose samples were used for immunohistochemical (IHC) analysis.

### Immunohistochemistry staining

**Fig. 2:**
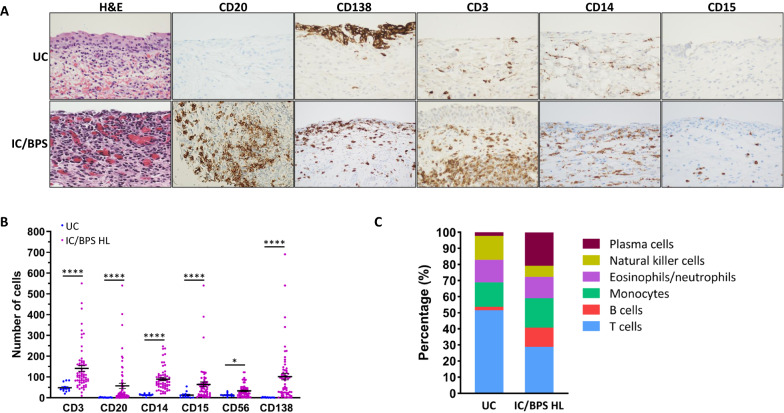
**A** Images of biopsied tissues from patients with Interstitial Cystitis/Bladder Pain Syndrome with Hunner Lesions (IC/BPS-HL) and unaffected controls (UC) stained for immune cell markers. Cross sections of bladder urothelium viewed at ×40 magnification and stained for Hematoxylin and Eosin (H&E, nucleus/cytoplasm), CD3 (pan T cells), CD20 (B cells), CD138 (plasma cells), CD14 (monocytes), and CD15 (neutrophils/eosinophils). Biopsies were performed on 48 patients diagnosed with IC/BPS-HL and 2 unaffected controls (UC). Thin (4–6 µm) cross sections of bladder tissue were then stained with either H&E or specific CD markers. Images of Hunner lesions and control tissue are shown. **B** Average cell marker counts found in the bladder biopsy samples of patients with IC/BPS-HL vs. UC. Cells positive for each marker were quantified per high-dry field (×400), taken in the area of maximum diffuse infiltration by the inflammatory cells. Data are expressed as mean ± SEM. **C** Relative proportions of cells displaying each cell marker in UC and IC/BPS-HL cohorts. The average proportion of Cluster of Differentiation (CD)-20 positive (CD20+) B cells (IC/BPS-HL: 12%; UC: 2%) and CD138+ plasma cells (IC/BPS-HL: 21%; UC: 2%) was much greater in pooled samples from patients with IC/BPS-HL compared to pooled samples from unaffected controls, while the percentage of Natural Killer cells (IC/BPS-HL: 7%; UC: 15%), Monocytes (IC/BPS-HL: 18%; UC: 15%), and Neutrophils (IC/BPS-HL: 14%; UC: 13%) did not differ significantly between the two cohorts. The relative abundance of cells displaying the CD3 T cell marker fell from 52% among the unaffected controls to 29% among the IC/BPS-HL cohort

The presence of HL was confirmed by cystoscopy prior to sample retrieval. Thin 4–6 µm tissue sections were cut and stained with Hematoxylin and Eosin (H&E). Using similar sections, IHC stains were performed on each biopsy sample (pooled into groups of 5 patients) for CD3, CD20, CD14, CD15, CD56, and CD138, for the quantification of T cells, B cells, monocytes, eosinophils/neutrophils, natural killer (NK) cells, and plasma B cells respectively (Fig. 2A). Hematoxylin was used as the counterstain. All antibodies were obtained from Ventana medical systems, Tucson, AZ. Antibody product details are as follows: CD3 (Catalog #: 790-4341, Lot F21480), CD20 (760-2531, Lot F18651) CD14 (760-4523, Lot V0001577) CD15 (760-2504, Lot F16036) CD56 (790-4465, Lot F13782) CD138 (760-4248, Lot 20065709).

After IHC staining, counting was performed and recorded per high-dry field (400×) in the area of maximum diffuse infiltration by the inflammatory cells. Areas of distinct lymphoid follicle or tight aggregate formation were not counted to prevent skewing of results. However, the presence of such lymphoid-predominant areas was noted. Biopsies from negative controls were examined and quantification of cells was performed along with the study cohort. Both staining and cell counting was conducted in a blinded manner. Multiple counts were recorded from each patient and the average cell count for each marker was used during data analysis.

### Mesoscale discovery cytokine assay

Levels of 9 different immunoregulatory cytokines (IFN-γ, IL-1Β, IL-2, IL-4, IL-6, IL-8. IL-12P70, IL-13, and TNF-α) were assessed using a Mesoscale Discovery (MSD) U-plex 10 spot multiplex assay (Mesoscale discovery, Catalog # K15049K-1). After preparing the 96-well plate as per manufacturer instructions, normalized urine samples from 18 IC/BPS-HL, 18 age and sex matched IC/BPS-NHL counterparts, and 4 UC research participants were tested in duplicate. Plate readings were conducted using an MSD Quickplex SQ 120 imager (Model no: 1250) and the data used for analysis was obtained using the MSD workbench software program (version 3.0.18).

All patients involved in the study were screened prior to selection to ensure none of them were diagnosed with any inflammatory comorbidities that could act as confounding variables and potentially skew the results of the IHC or cytokine analyses. In addition, all patients were also screened to ensure they were not taking any medications that could impact biomarker expression at the time of sample acquisition. Lastly, medical professionals from the same Urology clinic who have been treating these patients for many years and are familiar with their IC/BPS history gave final approval for the enrollment and subsequent sample collection of all patients enrolled in this study.

### Creatinine normalization

Creatinine normalization is utilized to prevent results from being influenced by a confounding factor such as patient hydration status at the time of sample donation. Creatinine is known to be excreted at a constant rate. Therefore, urinary creatinine concentration can serve as a tool to adjust for differences in urinary dilution among a heterogenous patient cohort. Multiple studies have confirmed the efficacy and impact of creatine normalization [16–19]. Many of these studies compared differences in urinary drug test results before and after creatinine normalization and concluded that Creatinine led to more accurate results [16, 18]. Considering this information regarding Creatinine’s use as an established normalization agent, urine from each patient was tested for Creatinine concentration in order to account for confounding variables. An Invitrogen Creatinine urinary detection kit (Thermofisher catalog number: EIACUN) was used and the manufacturer protocol was followed.

### Statistical analysis

Statistical analyses were performed using GraphPad Prism 9 and SPSS v27.0 (IBM, Armonk, NY). Data are presented as mean ± standard error of the mean (SEM). Comparisons among the groups were performed by the non-parametric Mann–Whitney U test. Ordinal logistic regression was performed to determine if increasing levels of urinary cytokines, B cells, or plasma cells were correlated with more severe clinical symptoms. Pearson’s correlation coefficients were calculated to determine any correlation between levels of CD3, CD20, CD14, CD15, CD56, and CD138 in bladder biopsies of IC/BPS patients with Hunner lesions.

### Clinical information

Clinical information, including average nocturia frequency per night, average quality of life, or average daily pain, was obtained from surveys responses. These surveys were either provided to the patients directly as a component of their participation in the study or retrospectively reviewed in the medical records.

## Results

### Patient biopsy and sample collection

Biopsy samples were acquired from the Hunner Lesions of 48 patients with IC/BPS-HL after obtaining informed consent. Biopsy samples from 2 patients who presented with benign bladder tumors and no morphologic or clinical features of IC or any other urological malignancy were also retrieved to serve as unaffected controls (UC). Biopsy samples from patients with IC/BPS-NHL were not obtained because patients without Hunner Lesions rarely undergo biopsy and IRB stipulations did not allow for biopsy collection if a biopsy was not already scheduled as standard of care.

Urine samples were obtained from 18 patients with IC/BPS-HL, 18 with IC/BPS-NHL and 4 UC. These samples were obtained from a separate patient cohort than the one whose samples were used for IHC analysis. Urine was first centrifuged to remove heavy debris and then normalized using a creatinine detection kit. Concentrations of urinary creatinine ranged from 11 to 279 mg/dL. Clinical data for all patients involved in the study is presented in Additional file 1: Tables S1 and S2.

### Immunohistochemistry of biopsied specimens from IC/BPS patients with and without Hunner lesions

In the present experiments, IHC was used to examine a diverse variety of immune cell markers in HL biopsies, including CD3 (T cells), CD20 (B cells), CD14 (monocytes/macrophages), CD15 (granulocytes including neutrophils and eosinophils), CD56 (natural killer cells), and CD138 (plasma cells) (Fig. 2A). On average, 481 cells expressing one of the immune cell markers were found in each of the IC/BPS-HL bladder samples compared to only 95 in the control samples (p < 0.0001). Average counts for cells displaying each individual marker of interest were significantly higher among patients with IC/BPS-HL vs. UC’s (Fig. 2B, C). Strikingly, CD138+ plasma cell counts displayed the largest discrepancies: a more than 50-fold increase in IC/BPS-HL samples (on average, 101 cells/field) as opposed to the UC cohort (2 cells/field; p < 0.0001; Fig. 2B). CD20+ B-cell counts were also elevated among the patients with IC/BPS-HL (57 cells/field) when compared to UC (2 cells/field; p < 0.0001). While immune cell concentrations were associated with the presence of disease, there was no significant correlation between increasing levels of B cells or plasma cells and disease severity symptoms such as average nocturia frequency per night, average quality of life, or average daily pain.

Other immune cell types were also observed to be more prevalent in the IC/BPS-HL samples than in controls. For T cells, the number of CD3+ cells were significantly elevated in patients with IC/BPS-HL (141/field) as opposed to UC (49 cells/field; p < 0.0001; Fig. 2B). Numbers of monocytes and macrophages, as reflected by CD14+ cells were also significantly elevated among the IC/BPS-HL cohort (89 cells/field) vs. the UC group (14 cells/field; p < 0.0001; Fig. 2B). Likewise, granulocytes including eosinophils and neutrophils, as reflected by CD15+ cell counts were more prevalent in samples from patients with IC/BPS-HL (65 cells/field) compared to UC (13 cells/field; p < 0.0001; Fig. 2B). Similarly, patients with IC/BPS-HL showed higher levels of CD56+ NK cells (33 cells/field) vs. UC (14 cells/field; p = 0.0249).

### Detection of cytokines present in the urine of IC/BPS patients with or without Hunner lesions

Urine cytokines in the present study were detected using the mesoscale discovery (MSD) U-plex assay: a highly sensitive electrochemiluminescence plate assay. Nine cytokines were selected for testing based on previous literature and their roles in the immune system: IFN-γ, TNF-α, IL-1β, IL-2, IL-4, IL-6, IL-8, IL-12p70, and IL-13.

**Fig. 3:**
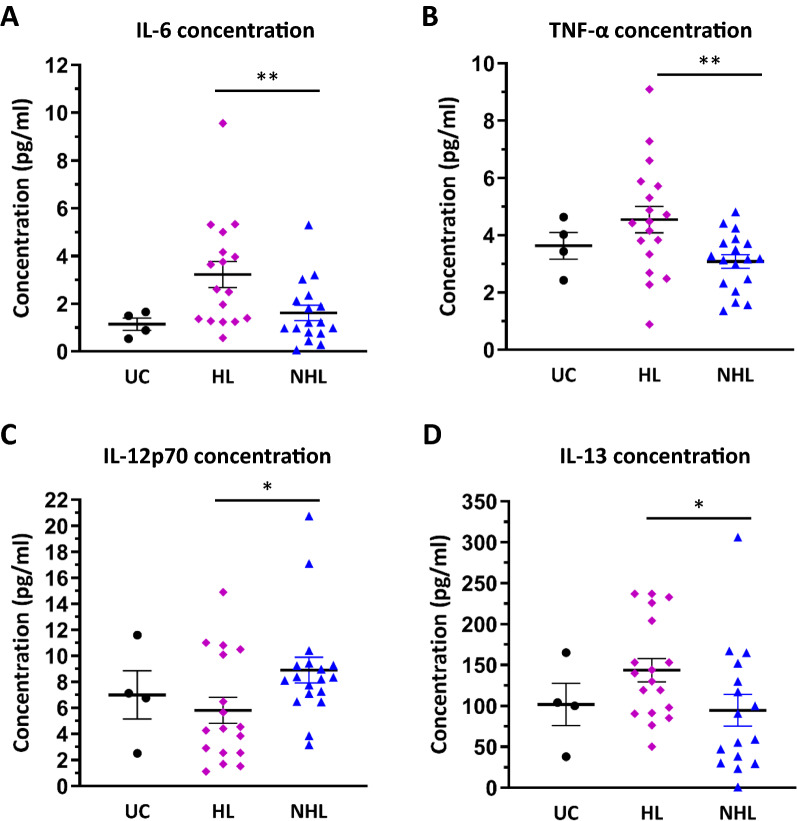
Urinary Cytokine concentrations in patients with Interstitial Cystitis/Bladder Pain Syndrome (IC/BPS), and unaffected controls (UC). Nine cytokines (Interferon-γ, Interleukin (IL)-1β, IL-2, IL-4, IL-6, IL-8, IL-12p70, IL-13, and Tumor Necrosis Factor-α) were assessed using a MSD U-plex 10 spot assay (Mesoscale discovery, Catalog # K15049K-1). Individual and average (indicated by the horizontal bar) cytokine concentrations for **A** IL-6, **B** TNF-α, **C** IL-12p70, and **D** IL-13 from patients with Interstitial Cystitis/Bladder Pain Syndrome without Hunner Lesions (IC/BPS-NHL), patients with Interstitial Cystitis/Bladder Pain Syndrome without Hunner Lesions (IC/BPS-HL), and UC research participants. Data are expressed as mean ± SEM

IL-6 is a key cytokine involved in B cell activation [20] and the results from the cytokine assay indicated that patients with IC/BPS-HL, when compared to their age and sex matched IC/BPS-NHL counterparts, displayed significantly higher average urinary concentrations of IL-6 (Fig. 3A; HL: 3.23 pg/mL; NHL: 1.61 pg/mL; p = 0.0054). A significant difference in average urinary IL-6 concentrations was not observed between the IC/BPS-HL population and UC population (Fig. 3A; HL: 3.23 pg/mL, UC: 1.14 pg/mL, P value = 0.0648).

TNF-α serves as a central mediator of the immune response and is produced by immune cells such as mast cells and fibroblasts [21]. Urinary TNF-α concentrations were also significantly elevated among the IC/BPS–HL cohort compared to the IC/BPS-NHL group (Fig. 3B; HL: 4.55 pg/mL; NHL: 3.08 pg/mL; p = 0.0064).

IL-12p70 is a pro-inflammatory cytokine that plays a key role in inducing T helper 1 (Th1) and Th17 T cells [22, 23]. Urinary IL-12p70 concentrations were significantly lower in the IC/BPS-HL population compared to the IC/BPS-NHL cohort (Fig. 3C; HL: 5.81 pg/mL; NHL: 8.91 pg/mL; p = 0.0422). There was no significant difference between UC and HL urinary IL-12p70 concentrations (Fig. 3C; HL: 5.81 pg/mL; UC: 7.00 pg/mL; p = 0.4528).

Urinary IL-13 concentrations were significantly higher in the IC/BPS-HL population compared to the IC/BPS-NHL cohort (Fig. 3D; HL: 143.66 pg/mL; NHL: 94.48 pg/mL; p = 0.0304). Urinary concentrations of IFN-γ, IL-1Β, IL-2, IL-4, and IL-8 did not yield statistically significant results in our patient cohorts. There was no statistically significant correlation between the concentration of any cytokines in the urine of patients and the severity of clinical symptoms such as urinary urgency or severity of pain.

### Correlation between different cell markers identified in patients with IC/BPS with Hunner lesions

Significant positive correlations were found between the following cell markers in biopsies of IC/BPS patients with Hunner lesions: CD20 and CD3, CD20 and CD14, CD20 and CD138, CD3 and CD14, CD14 and CD15, and CD14 and CD138 (p < 0.05) (Additional file 1: Table S3).

## Discussion

This study indicates that there are many significant differences in both the number of immune cells within the bladder and cytokine concentrations in the urine between patients with IC/BPS-HL and unaffected controls.

A particularly striking difference in patients with IC/BPS-HL was the significant elevation in numbers of cells expressing the B lineage CD20 and CD138 cell markers (Fig. 2A, B). This drastic increase in the proportion of CD20+ and CD138+ cells indicates that B and plasma cells may have a specific role to play in the development or pathology of Hunner lesions. This observation can serve as a starting point for identifying candidate mechanisms, diagnostic markers, or therapeutics for IC/BPS-HL [12]. These results are consistent with previous findings that also observed significantly increased CD20+ and CD138+ cell counts within the bladders of patients with IC/BPS-HL [12, 14, 15]. These previous studies typically analyzed 2–3 markers. The large number of immune cell markers analyzed in the present study allowed for the comparison of multiple cell types and therefore established that the relative increase in the number of B cells is especially noteworthy.

The results from the MSD cytokine assay are also concordant with the observation of increased numbers of B cells in patients with IC/BPS-HL. IL-6 is a key cytokine involved in B cell activation [20]. Urinary IL-6 concentrations were significantly higher in patients with IC/BPS-HL compared to patients with IC/BPS-NHL (Fig. 3A). These results suggest that IL-6 may play a role in the observed increased numbers of CD20+ and CD138+ cells in the bladder of patients with IC/BPS-HL. The results from this MSD assay replicate results from numerous previous studies that also found that urinary IL-6 levels were elevated in patients with IC/BPS-HL [9, 24]. While IL-6 elevation is not specific for IC/BPS-HL [25], and the small difference between UC and patients with IC/BPH-HL may make the design of a clinical test difficult, our observation of increased IL-6 levels is consistent with B cell infiltration into the bladder and provides an important piece of evidence about the immunology of the disease.

The IHC results indicate that, when compared to UCs, patients with IC/BPS-HL also displayed significantly higher numbers of CD3+ T cells within their bladders (Fig. 2B; HL: 141, UC: 49; p < 0.0001). Interestingly, urinary IL-12p70 concentrations were significantly lower among the IC/BPS-HL sub population compared to the IC/BPS-NHL cohort (Fig. 3C; HL: 5.81 pg/mL; NHL: 8.91 pg/mL; p = 0.0422). IL-12p70 is a pro-inflammatory cytokine that plays a key role in inducing Th1 and Th17 T cells [22, 23]. There was no significant difference between UC and HL urinary IL-12p70 (Fig. 3C; HL: 5.81 pg/mL; UC: 7.00 pg/mL; p = 0.4528). Additional studies will be necessary to determine if the elevation of IL-12p70 is consistently present in patients with IC/BPS-NHL. Given the relationship between IL-12p70 and T cell activation pathways, further testing could focus on confirming the specific T cell subsets that are upregulated among patients with IC/BPS-NHL and -HL. For instance, further analysis of the types of immune cells involved in IC/BPS-NHL could help determine if these patients have greater activation of Th1 and Th17 cells than patients with IC/BPS-HL.

Significant differences between the IC/BPS-HL and IC/BPS-NHL cohorts were also observed in urinary TNF-α levels (Fig. 3B; HL: 4.55 pg/mL; NHL: 3.08 pg/mL; p = 0.0064). The increased urinary TNF-α concentrations found amongst the IC/BPS-HL cohort builds upon previous findings which describe significantly elevated levels of TNF-α in the sera of patients with IC/BPS and identify the cytokine as a possible mediator of bladder urothelium apoptosis, which is observed in IC/BPS [26, 27]. As TNF-α is increased in overactive bladder as well as IC/BPS [21], measurements of TNF-α concentration on their own will be unlikely to produce a stand-alone clinical test for IC/BPS. However, the consistent observation of increased TNF-α in patients with IC/BPS will be useful for establishing accurate and informative immunological models.

While we used an expanded panel of immune markers, the relative increase in B cells and plasma cells in IC/BPS patients with Hunner lesions observed in this study are consistent with prior findings in the field [12, 15]. Given the increased proportion of plasma cells found in the bladder of patients with IC/BPS and strong association between Toll-Like Receptor (TLR) activity and IC/BPS severity, [28] it is possible that TLR activity mediated through a B cell activation pathway is involved in IC/BPS pathogenesis. TLR’s are involved in T cell independent B cell activation, and increased TLR-4 signaling has been associated with increased pain and symptom severity among women with IC/BPS, and other inflammatory pain conditions, including rheumatoid arthritis. In patients with IC/BPS, elevated TLR activity correlates with increased pain frequency, pain intensity, higher rates of reduced sexual functioning, and overall higher Genitourinary Pain Index scores [28]. In vitro stimulation of B cells by TLR’s has been shown to induce B cell proliferation and differentiation into activated antibody-producing plasma cells and in vivo TLR signaling contributes to T cell independent antibody responses to bacterial infection [29]. Thus, it is possible that the increased numbers of B cells and plasma cells we detected in the bladder of IC/BPS patients is due to the activation of TLR-4.

Our results indicate that activation of B-cells and plasma cells correlates with IC/BPS-HL disease status but we did not find any correlation between cell concentrations and severity of specific symptoms. This conclusion is based on the high number of CD138+ and CD20+ cells found in the bladder tissue and the significant increase in IL-6 urinary concentrations among the IC/BPS-HL cohort. This points to a role of B cell maturation in IC/BPS-HL. While our results strongly indicate B cell involvement, the study design cannot determine whether the presence of these cells is a consequence or a cause of disease. It is likely that the increase in immune cell counts and coinciding upregulation of certain pro inflammatory cytokines represent a disease process in IC/BPS, but we cannot exclude that their presence may be neutral or even protective. Future studies should address the relationship between immune cell activation and IC/BPS disease severity to establish a causal relationship.

We did not find a statistically significant correlation between the concentration of cytokines in the urine of patients and the severity of clinical symptoms such as urinary urgency or severity of pain. The interpretation of this result is complicated because we cannot be certain whether it is due to natural variability in the levels of cytokines between different people or a true lack of a relationship between cytokine levels and disease. Longitudinal analysis of the relationship between clinical symptoms and degree of inflammatory changes can help resolve this issue.

It is still unclear how chemokines and cytokines in urine or serum correlate to bladder immune infiltration. Since chemokines and cytokines are indicators of inflammation, exploring the relationship between them and bladder immune cells in IC/BPS should be a high priority for future research. In the present study, biopsy samples were collected asynchronously with urine samples, so a correlation analysis could not be performed. While it is not always technically or ethically possible to collect urine and biopsy specimens from a given patient simultaneously, it is a direction that should strongly be considered in future studies. These future studies should also include more control patients when ethically feasible to improve the strength of comparison between the IC/BPS and control cohorts.

### Future directions

The enrichment of B cells within HL raises the question of what role these cells play in disease. While a cause-and-effect relationship may not be established from a case–control study, such as the present work, multiple additional experiments could help clarify the role of B and plasma cells in IC/BPS. For example, sequencing the B cell receptors found within HL can help clarify whether there is a certain antigen or group of antigens to which the B cells are responding. Once an antibody or antibodies produced by B cells in multiple patients is identified, an antigen library could be screened to identify the molecule to which the antibodies bind. If the molecule is bacterial, viral or fungal, it is possible that the etiology of IC/BPS-HL is partially infectious; and if the antigen is normally found in bladder cells, there is an autoimmune component to IC/BPS-HL. Depending on the nature of the antigen, it is possible to design interventions to target the presumed nature of the disease in clinical trials. For example, an antigen of bacterial origin would strongly indicate that antibiotic therapy may be indicated. In contrast, if the target of the B cells is a protein produced by bladder cells, treatments for autoimmune disease, such as biologics, may be useful to reduce disease burden for these patients.

## Supplementary Information

**Additional file 1: Table S1.** Clinical characteristics and sex breakdown of the 3 research cohorts whose urine was collected for analysis with the Mesoscale discovery (MSD) multiplex cytokine assay. Proper informed consent was obtained from all patients prior to sample retrieval. (IPSS: International Prostate Symptom Score; UC: Unaffected controls; NHL: Patients with Interstitial Cystitis/Bladder Pain Syndrome without Hunner Lesions; HL: Patients with Interstitial Cystitis/Bladder Pain Syndrome with Hunner Lesions). **Table S2.** Clinical characteristics and sex breakdown of the 2 research cohorts who donated biopsies for immunohistochemistry analysis of bladder tissue samples. Proper informed consent was obtained from all patients prior to sample retrieval. Since UC patients did not present with urological symptoms, they were not asked about clinical information regarding their voiding and urinary associated pain, and therefor this information was not available in their clinical records. (UC: Unaffected controls; IC/BPS-HL: Patients with Interstitial Cystitis/Bladder Pain Syndrome with Hunner Lesions). **Table S3.** Correlation between cell markers in bladder biopsies of IC/BPS patients with Hunner lesions. Immune cell correlation calculated by Pearson’s correlation coefficients between levels of CD3, CD20, CD14, CD15, CD56, and CD138 in bladder biopsies of IC/BPS patients with Hunner lesions. Significant positive correlations were found between the following cell markers in biopsies of IC/BPS patients with Hunner lesions: CD20 and CD3, CD20 and CD14, CD20 and CD138, CD3 and CD14, CD14 and CD15, and CD14 and CD138 (p < 0.05).

## Data Availability

All data and patient information is stored in secure cloud-based storage drives and can be made available upon request.
