# Supplementary material for: Immune cell profiles of patients with interstitial cystitis/bladder pain syndrome
Source: J Transl Med. 2022 Feb 21;20:97. doi: 10.1186/s12967-022-03236-7 (PMC8862517; doi:10.1186/s12967-022-03236-7)
Supplement: Supplementary file 1 — Additional file 1: Table S1. Clinical characteristics and sex breakdown of the 3 research cohorts whose urine was collected for analysis with the Mesoscale discovery (MSD) multiplex cytokine assay. Proper informed consent was obtained from all patients prior to sample retrieval. (IPSS: International Prostate Symptom Score; UC: Unaffected controls; NHL: Patients with Interstitial Cystitis/Bladder Pain Syndrome without Hunner Lesions; HL: Patients with Interstitial Cystitis/Bladder Pain Syndrome with Hunner Lesions). Table S2. Clinical characteristics and sex breakdown of the 2 research cohorts who donated biopsies for immunohistochemistry analysis of bladder tissue samples. Proper informed consent was obtained from all patients prior to sample retrieval. Since UC patients did not present with urological symptoms, they were not asked about clinical information regarding their voiding and urinary associated pain, and therefor this information was not available in their clinical records. (UC: Unaffected controls; IC/BPS-HL: Patients with Interstitial Cystitis/Bladder Pain Syndrome with Hunner Lesions). Table S3. Correlation between cell markers in bladder biopsies of IC/BPS patients with Hunner lesions. Immune cell correlation calculated by Pearson’s correlation coefficients between levels of CD3, CD20, CD14, CD15, CD56, and CD138 in bladder biopsies of IC/BPS patients with Hunner lesions. Significant positive correlations were found between the following cell markers in biopsies of IC/BPS patients with Hunner lesions: CD20 and CD3, CD20 and CD14, CD20 and CD138, CD3 and CD14, CD14 and CD15, and CD14 and CD138 (p < 0.05). [file 12967_2022_3236_MOESM1_ESM.docx]

**Additional file 1**


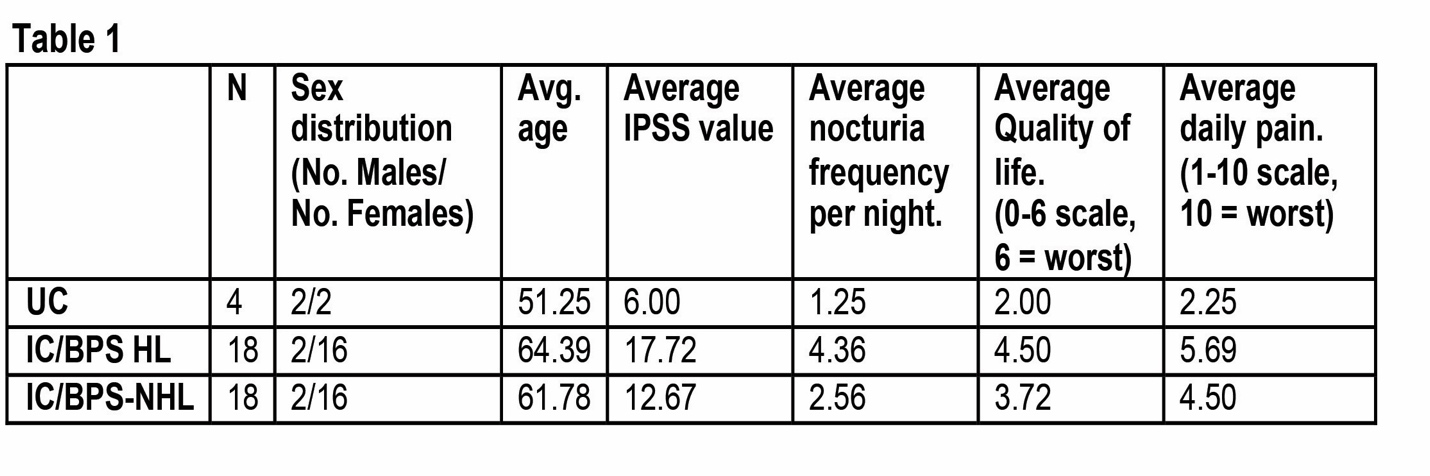


**Table S1:** Clinical characteristics and sex breakdown of the 3 research cohorts whose urine was collected for analysis with the Mesoscale discovery (MSD) multiplex cytokine assay. Proper informed consent was obtained from all patients prior to sample retrieval.

(IPSS: International Prostate Symptom Score; UC: Unaffected controls; NHL: Patients with Interstitial Cystitis/Bladder Pain Syndrome without Hunner Lesions; HL: Patients with Interstitial Cystitis/Bladder Pain Syndrome with Hunner Lesions).


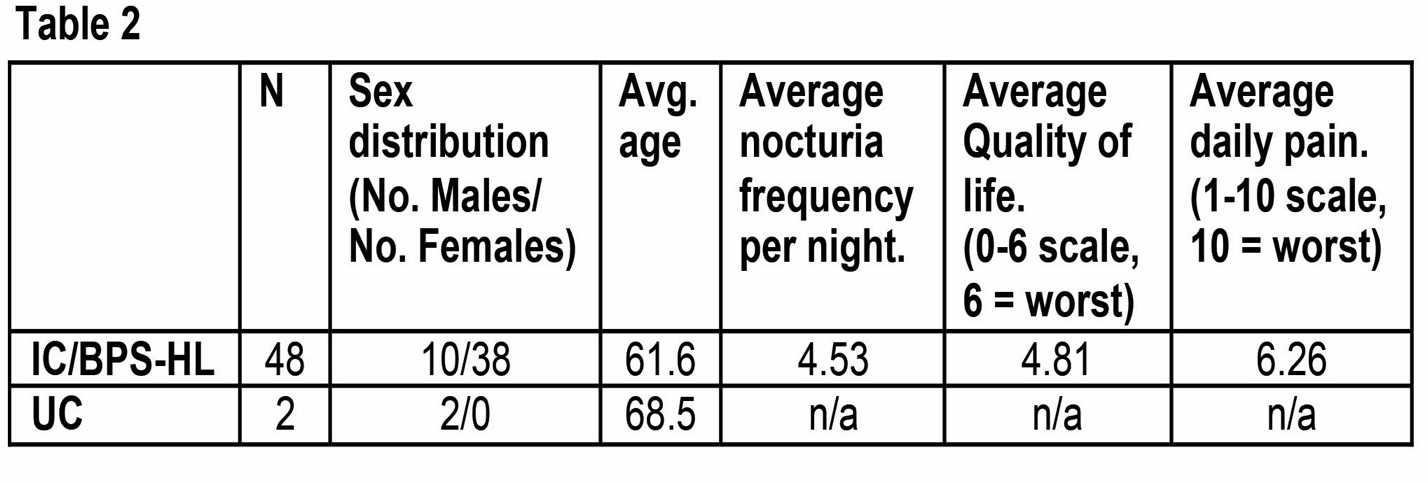


**Table S2:** Clinical characteristics and sex breakdown of the 2 research cohorts who donated biopsies for immunohistochemistry analysis of bladder tissue samples. Proper informed consent was obtained from all patients prior to sample retrieval. Since UC patients did not present with urological symptoms, they were not asked about clinical information regarding their voiding and urinary associated pain, and therefor this information was not available in their clinical records. (UC: Unaffected controls; IC/BPS-HL: Patients with Interstitial Cystitis/Bladder Pain Syndrome with Hunner Lesions)

|  |  | CD56 | CD20 | CD3 | CD14 | CD15 | CD138 |
| --- | --- | --- | --- | --- | --- | --- | --- |
| CD56 | Pearson Correlation | 1 | 0.237 | -0.21 | 0.213 | 0.050 | -0.019 |
|  | Sig. (2 tailed) | - | 0.112 | 0.892 | 0.159 | 0.742 | 0.903 |
|  | N | 46 | 46 | 46 | 45 | 46 | 45 |
| CD20 | Pearson Correlation | 0.237 | 1 | 0.470* | 0.523* | -0.073 | 0.295* |
|  | Sig. (2 tailed) | 0.112 | - | <0.001 | <0.001 | 0.624 | 0.046 |
|  | N | 46 | 47 | 47 | 46 | 47 | 46 |
| CD3 | Pearson Correlation | -0.021 | 0.470* | 1 | 0.313* | 0.164 | 0.148 |
|  | Sig. (2 tailed) | 0.892 | <0.001 | - | 0.034 | 0.271 | 0.327 |
|  | N | 46 | 47 | 47 | 46 | 47 | 46 |
| CD14 | Pearson Correlation | 0.213 | 0.523* | 0.313* | 1 | 0.438* | 0.450* |
|  | Sig. (2 tailed) | 0.159 | <0.001 | 0.034 | - | 0.002 | 0.002 |
|  | N | 45 | 46 | 46 | 46 | 46 | 45 |
| CD15 | Pearson Correlation | 0.050 | -0.073 | 0.164 | 0.438* | 1 | 0.062 |
|  | Sig. (2 tailed) | 0.742 | 0.624 | 0.271 | 0.002 | - | 0.683 |
|  | N | 46 | 47 | 47 | 46 | 47 | 46 |
| CD138 | Pearson Correlation | -0.019 | 0.295* | 0.148 | 0.450* | 0.062 | 1 |
|  | Sig. (2 tailed) | 0.903 | 0.046 | 0.327 | 0.002 | 0.683 | - |
|  | N | 45 | 46 | 46 | 45 | 46 | 46 |

All samples were obtained from IC/BPS patients with Hunner lesions.

*Correlation is significant at p <0.05.

**Table S3:** Correlation between cell markers in bladder biopsies of IC/BPS patients with Hunner lesions. Immune cell correlation calculated by Pearson's correlation coefficients between levels of CD3, CD20, CD14, CD15, CD56, and CD138 in bladder biopsies of IC/BPS patients with Hunner lesions. Significant positive correlations were found between the following cell markers in biopsies of IC/BPS patients with Hunner lesions: CD20 and CD3, CD20 and CD14, CD20 and CD138, CD3 and CD14, CD14 and CD15, and CD14 and CD138 (p<0.05).
